# A Flow Sensing Device Formed Exclusively by Employing Additive Manufacturing for On-Site Fabrication Aboard a Ship

**DOI:** 10.3390/s23208481

**Published:** 2023-10-15

**Authors:** Dimitrios-Nikolaos Pagonis, Ioannis Matsoukas, Grigoris Kaltsas, Aggelos Pilatis

**Affiliations:** 1 Naval Architecture Department, University of West Attica, 12243 Athens, Greece; na15058@uniwa.gr (I.M.); apilatis@uniwa.gr (A.P.); 2 microSENSES Laboratory, Electrical & Electronic Engineering Department, University of West Attica, 12243 Athens, Greece; gkaltsas@uniwa.gr

**Keywords:** additive manufacturing, 3D printing, 4D printing, FDM technology, sensors, spare parts, marine industry, vortex shedding

## Abstract

This work concerns the design, fabrication, and testing of a novel air-flow sensor employing exclusively additive manufacturing that can be fabricated on-site, aboard a ship, or in a similarly remote area, without relying on external manufacturing facilities. The developed device’s principle of operation is based on vortex shedding; its novelty focuses on employing solely additive manufacturing technology, for the manufacturing—in a single process step—of all the sensor’s main elements. In more detail, the required flow-shaping housing, the appropriate piezoresistive sensing element, and the electrical interconnection pads are all constructed in a single process step, through standard Fused Deposition Modeling (FDM) 3D technology. Direct communication to the necessary readout circuitry can be easily achieved through standard soldering utilizing the integrated contact pads of the sensor. The prototype was preliminary characterized, validating its proper functionality. Key features of the proposed device are low cost, fast on-site manufacturing of the entire measuring device, robustness, and simplicity, suggesting numerous potential applications in the shipbuilding industry and other industrial sectors.

## 1. Introduction

Additive manufacturing (3D printing) is a rapidly evolving process that is continuously emerging into a broad range of industrial and non-industrial applications. Currently, it is widely employed in fields such as aerospace, military, medicine surgical planning [1], art, food technology, and many other disciplines [2]. Furthermore, additive manufacturing draws large attention regarding health and medical-related applications (fabrication of vascular networks [3], tissue engineering scaffolds [4], exoskeletons [5], etc.). With respect to industrial applications, the specific process has the potential to revolutionize manufacturing industry and transform the production line; the main reasons supporting this potential are the continuously expanding list of available materials that can be adopted for manufacturing, the ability to construct complex objects, and on-site manufacturing—all opening new opportunities. In addition, over the last decade, a promising and rapidly evolving new discipline of additive manufacturing has emerged, known as 4D printing. By combining 3D printing techniques with novel building materials and appropriate design, 4D printing enables the creation of a printed object that can alter its geometry over time in response to its environment [6]. That is, through the use of 4D printing technology, the initial manufacture and final form of a part can be different, enabling each to be optimized for a different purpose. We should note that 4D printing has the potential to be employed for stimuli-responsive devices [7], a feature which is essential for the development of sensing devices.

Additive manufacturing has already been deployed successfully in industry, even in a prototype development level. In more detail, in the shipbuilding industry, Wärtsilä Corp, a global leader in smart technologies and complete lifecycle solutions for the marine and energy markets [8], have been employing carbon-fiber 3D printers (Markforged Co., Waltham, MA, USA) since mid-2018 to manufacture several tools for their factories. According to Markforged Co [9], Wärtsilä usually manufactures its lifting tools from solid steel, but the resulting tools are too expensive and time-intensive to manufacture, and too heavy for people to use and transport—an important drawback for the shipbuilding industry especially, where the service areas are practically distributed across the globe. In order to overcome the specific issues, a 3D printed CE-certified lifting tool was successfully manufactured, able to lift a 240 kg engine piston. The specific case study clearly shows the path of adopting widely additive manufacturing in the maritime industry sector. Therefore, we can safely conclude that additive manufacturing already has the potential to evolve into an efficient alternative manufacturing process for the production of large-scale, complex objects in shipbuilding.

It is important to note that the proper operation of all modern vessels relies heavily on the information provided by the onboard measuring devices that quantify critical performance parameters. During the past three decades, miniaturization of sensors has led to the newly developed field of “microsensors”—an emerging field that is growing rapidly, building on the vast advances of the semiconductor industry. As a consequence, sensors deployed in ships followed the same trend, taking their role one step further by incorporating semiconductor-based technology to standard marine equipment and measuring systems [10]. Currently, it is well accepted that modern sensing devices have a critical role in the shipbuilding industry for various applications. The typical type of sensors employed are gas-detection sensors, gas/air-flow sensors, humidity sensors, temperature and pressure sensors, speed and acceleration sensors, strain sensors etc., which are all essential with regard to vessels’ safety. As a common example, a typical Ro-Ro vessel has four medium power four-stroke engines installed onboard, which are required for the operation of the corresponding generator sets [11]. A possible failure even at only one of the installed internal combustion engines—not detected by the appropriate sensing device—will immediately result in a safety compromise, according to the corresponding safety regulations [12], with severe consequences.

Modern sensors that employ semiconductor technology, however, also present some drawbacks; that is, a complicated manufacturing process that requires expensive and special manufacturing facilities (clean room), an almost impossible customer customization/modification of the developed process steps for altering the device’s operational characteristics, expensive sensor packaging, indirect electrical communication between the sensing integrated circuit and the necessary readout circuitry, and high prototype-fabrication costs. Finally, on-site manufacturing of the sensing device is obviously not feasible.

The main innovation in the current research work is the development of a new state-of-the-art manufacturing procedure for the development of a novel monolithic sensing device employing only 3D printing for its on-site fabrication. The device’s principle of operation is based on vortex shedding, a well-studied phenomenon that is employed in various flow-measurement sensors [13], and describes the development of vortex shedding when an obstacle of appropriate geometry (Bluff Body) is placed in a flowing fluid. The main sensing element of the proposed device is inherently connected to the macroworld, overcoming the necessity for performing wire bonding (a delicate process for the creation of the necessary electrical pathways between microscale sensing elements and the corresponding macroworld circuitry which can be prone to damage), with immediate benefits in device complexity, mechanical reliability, process time, and cost [14,15]. We should note that the device can be manufactured employing a commercially available desktop 3D printer fast, on-site, and on demand, contributing to the reduction in the extent of the required stock-keeping onboard according to the corresponding regulations, regarding safety critical equipment/spare parts (Section 10.3, ISM Code) for vessels [16]. Low cost, robustness, and simplicity, which are the key features for the proposed device, suggest numerous potential industrial applications which obviously are not limited to the maritime sector.

## 2. Materials and Methods

### 2.1. Proposed Device’s Geometry and Principle of Operation

**Figure 1:**
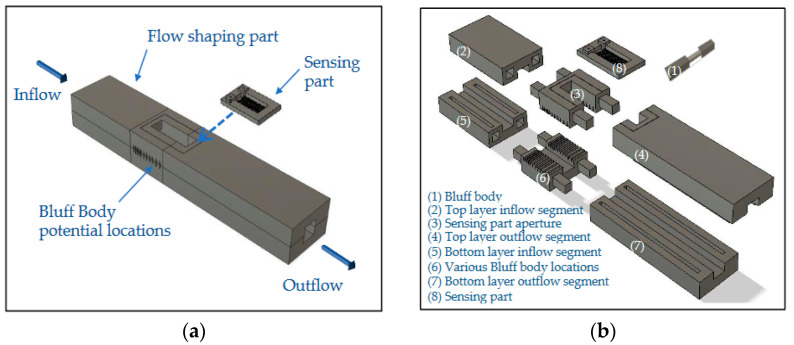
Schematic design of the proposed device: (**a**) 3D illustration; (**b**) exploded view.

**Figure 2:**
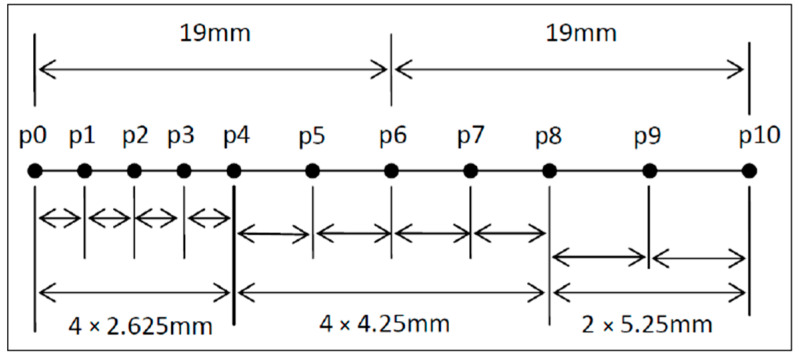
Possible Bluff Body locations along the flow stream, in the flow-shaping part of the proposed device [19].

The first developed sensor based on Karman vortex street was reported over 60 years ago [17], and it was employed successfully for measuring the speed of a vessel. The proposed device’s structure is similar to a typical vortex shedding flowmeter; it comprises two main parts, a flow shaping part, responsible for the development of vortex shedding, and a sensing part, designed to detect the formation of the Karman vortex street. Τhe principle of operation of the device can be briefly described as follows: A Bluff Body of suitable geometry is inserted into a rigid airflow channel (flow-shaping part), leading to the formation of a Karman vortex street in the presence of an appropriate airflow. The induced pressure evolution behind the Bluff Body—which varies accordingly to the airflow value—is measured through a piezoresistive element situated in the sensing part of the device. We should note that the geometry of the flow-shaping section is based on previous research on the development of a pneumatic energy generator using Karman vortex street [18], and CFD parameterization [19] of its geometry considering a fixed air velocity of 20.7 m/s. The purpose of this CFD parametric study was to understand the mechanism and the roles of the involved parameters in order to enhance the vortex shedding phenomenon. According to the main conclusions of the specific study, the shape of the Bluff Body is important in order to achieve significant pressure variations; moreover, the design of the entire flow-shaping configuration (i.e., the number of Bluff Bodies employed, their arrangement and the chosen blockage ratio) is crucial. The schematic design of the proposed device is presented in Figure 1; a rectangular flow channel is employed, while on the top side of the channel, a suitable aperture has been formed where the sensing part of the sensor is to be mounted on. We should note that the exact location of the Bluff Body at the area under the sensing part has a direct effect on the resulting developed pressure—induced by vortex street—that will be measured by the piezoresistive element. In addition, an appropriate combination of two Bluff Bodies that are placed in a series along the flow stream can result in a larger evolution of the induced pressure compared to employing only one [19]. For the above reasons, eleven possible Bluff Body locations were considered, in accordance with the relevant CFD study [18], in the flow-shaping part of the device, as presented in Figure 1 and Figure 2.

Note that positions p0 and p10 are oriented towards the inlet and outlet of the flow-shaping part accordingly, while appropriate apertures have been designed at the side-walls of the flow channel, in the area under the sensing part, as shown in Figure 1b in order for the Bluff Body (or Bodies) to be easily repositioned at a different location.

### 2.2. Flow-Shaping Part Design—Building Material Employed

**Figure 3:**
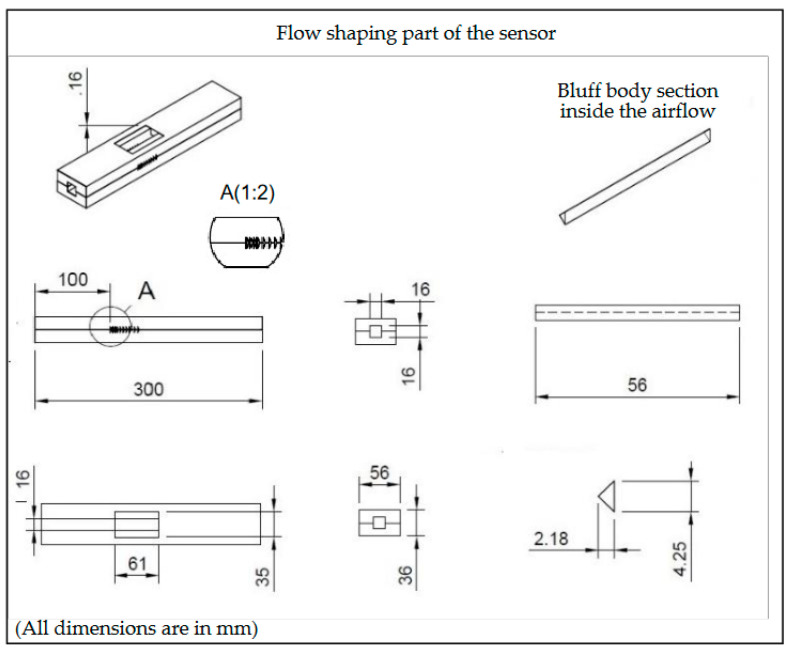
Design of the flow-shaping part of the sensor, responsible for the development of vortex shedding.

The design of the flow-shaping part of the sensor is presented in Figure 3. The flow channel has a cross-section area of 16 × 16 mm^2^ and a length of 300 mm, while the Bluff Body employed is an isosceles triangle (its dimensions are presented in the same figure), installed in a way that its base faces the incoming flow for maximum induced pressure evolution due to the formation of the Karman vortex street [18]. The building material selected for the construction of the flow-shaping section was BASF Acrylonitrile Styrene Acrylate (ASA) [20]. The specific material was selected due to its high temperature resistance compared to the traditional Polylactic Acid (PLA) widely employed. In more detail, ASA is a high-performance thermoplastic with similar mechanical properties as Acrylonitrile Butadiene Styrene (ABS), while its glass transition temperature is 112 °C, a feature essential for the formation of the piezoelectric element, as discussed in the next section.

### 2.3. Sensing Part Design—Building Material Employed

**Figure 4:**
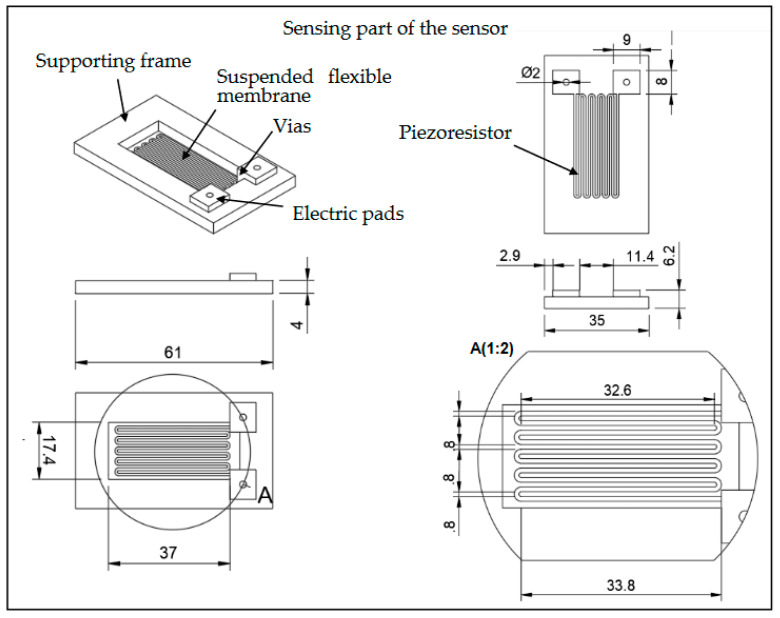
Schematic of the sensing part of the sensor, responsible for the detection of the pressure evolution due to the development of vortex shedding. Note that the piezoresistive sensing element is integrated on top of the flexible membrane which is supporting by a rigid frame; all elements are printed in a single step.

The sensing part of the device consists of three main subparts as presented in Figure 4. In more detail, it comprises a thin suspended membrane which is aligned with the airflow channel’s upper wall, an appropriate piezoresistor that is integrated on top of the membrane, and a rigid frame supporting the membrane. In order to maximize its sensitivity [21], the piezoresistive element has a typical meander geometry with the maximum number of grid lines (10) and length (32.6 mm), which can be safely printed using additive manufacturing, for the specific building material and the available area. The width of each line and the distance between two lines are both equal to 0.8 mm, while its thickness is set at 0.8 mm. We should note that the two piezoresistor ends—situated at the same side of the membrane—extend vertically from the membrane’s top surface, up to the level of the rigid frame, forming the necessary vias in order for the piezoresistor to be electrically connected to the necessary readout circuitry, through appropriate electric pads.

It is important to highlight two key characteristics concerning the specific part of the sensor. Firstly, the membrane has a noticeably smaller thickness than the channel’s walls (0.2 mm compared to 10 mm) in order to be adequately flexible. In this way, by embedding it into the flow channel, the membrane—acting like a diaphragm—deflects when evolution of pressure manifests itself due to the incoming flow. Secondly, all the elements comprising the sensing part are printed through additive manufacturing in a single step; that is, the rigid frame supporting the diaphragm, the piezoresistor, the vias, the electric pads, and the diaphragm itself consist a single printed part.

### 2.4. Fabrication of the Prototype Sensor Device

Both the flow-shaping and the sensing parts of the developed prototype were fabricated through standard Fused Deposition Modeling (FDM) 3D technology employing the commercially available desktop ULTIMAKER S3 printer [22]. According to the specific printer’s specifications, the minimum layer height (minimum thickness of each individual layer printed) is 20 μm, while the XYZ resolution (stepper motor positioning accuracy) is 6.9 μm, 6.9 μm, and 2.5 μm for each axis, respectively. It is worth noting that each part was printed in a single step.

**Figure 5:**
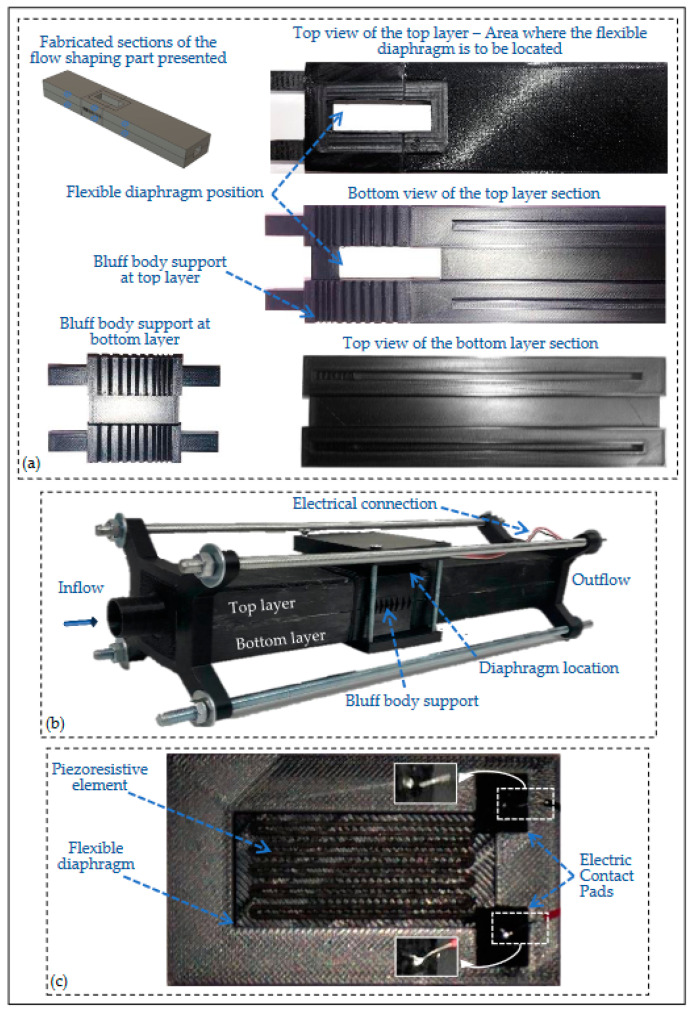
(**a**) Disassembled main sections of the flow-shaping part of the device—the corresponding schematic designs for the presented sections in Figure 1b are 3, 4, 6, and 7; (**b**) the complete prototype device—each part (shaping and sensing) is fabricated in a single step employing Fused Deposition Modeling (FDM) 3D technology; (**c**) the fabricated sensing part of the device.

The sensing part, main sections of the flow-shaping part, and the resulting assembled device are presented in Figure 5. Concerning the building materials for the fabrication of the sensing part, two filaments were employed: FiberForce Nylforce CNT Conductive (Treviso, Italy) for the conductive elements [23] and BASF (San Bruno, CA, USA) Acrylonitrile Styrene Acrylate (ASA) [20] for the remaining elements, i.e., the membrane together with its supporting frame. We should note that the FiberForce Nylforce CNT Conductive filament was selected due to its noticeably higher conductivity, compared to the other commercially available conductive filaments, according to its manufacturer [23]. The main characteristics for the conductive filament and the printing parameters for both materials employed are presented in Table 1 and Table 2, respectively.

According to the manufacturer of the conductive filament, the specific material consists of a biopolymer compound which is enriched with Carbon Nanotubes (CNs). We should note that Graphene and Carbon Nanotubes, are widely used in printed strain sensors employing inkjet or screen-printing technology because of their excellent electrical conductivity and mechanical reliability [24], while nanostructure engineered thermoplastic composites processed via additive manufacturing have been reported to feature a piezoresistive response [25]. Thus, it was the authors’ belief that the presence of the CNs in a commercially available 3D building material would also result to a similar piezoresistive response for the final printed element, enabling the detection of the membrane deformation due to the presence of the incoming flow, an assumption that is clearly confirmed by the experimental data presented in Section 3. Furthermore, an important characteristic of the resulting device is the direct electrical communication between the sensing element and the necessary readout circuitry that can be easily achieved through standard soldering on the integrated (printed) electric contact pads.

## 3. Results

### 3.1. Thermal Treatment of the Piezoresistive Element

The idea of directly printing a functional pattern on a flexible or rigid substrate is not new; for example, some of the earliest uses of inkjet printing of a conductive material originated in the 1990s [26]. Currently, conductive nanoparticle-based inks are being employed in order to create inkjet-printed sensing devices with fabricated patterns such as microheaters [27], strain gauges [28], and other key elements. In order to decrease the resulting electrical resistance of these printed patterns, however, a post-printing sintering step is necessary that usually requires thermal treatment at a temperature of more than 200 °C [29]. Therefore, employing FDM additive manufacturing technology in order to create a conductive element using a CN-enriched filament poses a constraint concerning the sintering step since 3D filaments do not have adequate heat resistance. For example, the melting point of the conductive building material employed (FiberForce Nylforce CNT Conductive) is 145–160 °C [23] since its heat resistance is limited by the presence of the biopolymer compound; obviously, the same applies for the other types of commercially available polymer-based conductive filaments where Polylactic Acid (PLA), Acrylonitrile Butadiene Styrene (ABS), or Thermoplastic Polyurethane (TPU) is the base material [30,31,32]. Nevertheless, the mechanism of sintering relies not only on the temperature employed since other factors are also dominant, such as conductive particle size and shape, printed pattern thickness, and time [33]. Furthermore, although the applied temperature clearly has a significant effect on the degree of sintering, the time applied also significantly affects the outcome [34]. For the above reasons, the effect of thermal sintering, employing a low temperature of 100 °C, on the resulting electrical resistance of the conductive 3D-printed element was investigated.

The particular investigation was performed as follows: The fabricated sensing part of the sensor (Figure 5c) was heated at a temperature of 100 °C for a given number of two-hour time intervals, while its electrical resistance was measured through a Keithley 2401 (Solon, OH, USA) source meter at each time step. We should note that the manufacturer of the specific filament suggests a post-printing crystallization through thermal annealing in the range of 145–160 °C for obtaining a 100 °C heat resistance of the printed structure. Nevertheless, a higher sintering temperature was not selected since the conductive pattern is printed concurrently with the non-conductive elements of the sensing part, i.e., the diaphragm and its supporting frame, in one step. Although the building material selected for printing all the non-conductive elements of the sensor is BASF Acrylonitrile Styrene Acrylate (ASA), which is a high-performance thermoplastic with similar mechanical properties to Acrylonitrile Butadiene Styrene (ABS) and a higher heat resistance compared to the traditional Polylactic Acid (PLA) widely employed, its glass transition temperature is 112 °C [20], limiting the temperature for the thermal treatment of the sensing part.

**Figure 6:**
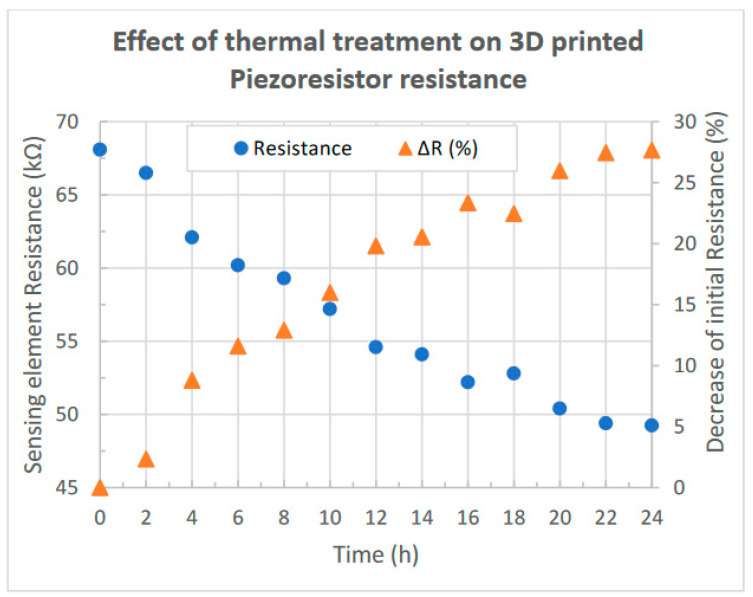
Effect of thermal sintering on the electrical resistance of the 3D-printed piezoresistor; an almost 30% decrease in the initial value is noticed.

The measured electrical resistance of the conductive element together with the corresponding decrease as a percentage of its initial value for each sintering step performed are presented in Figure 6. As we can see, the initial resistance value of 68.1 kΩ was reduced to 49.25 kΩ after 24 h of thermal sintering; thus, a significant total decrease of approximately 28% of the initial value is obtained after performing twelve steps of two-hour thermal sintering at a temperature of 100 °C. From the above results, we can safely assume that thermal sintering has an important role in the obtained resistance of a conductive pattern which is fabricated employing FMD 3D technology, in correspondence with the cases of inkjet printing or screen-printing. Furthermore, due to the limitation imposed by the building materials on the temperature, a significantly larger treatment time is required.

### 3.2. Experimental Setup Employed for the Characterization of the Sensor

**Figure 7:**
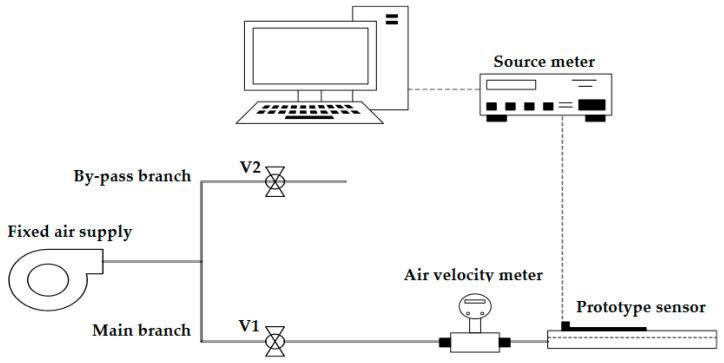
Experimental setup employed for the initial characterization of the developed sensor. It mainly consists of an appropriate flow network, a centrifugal fan, a source meter, and an air-velocity meter.

An initial electrical characterization of the developed sensor was performed employing the experimental setup presented in Figure 7; it mainly consists of an industrial 0.75 kW centrifugal fan with a fixed air supply of 3400 LPM, an air-velocity measuring unit, an appropriate flow network, two needle valves, and a Keithley 2401 source meter. The flow network comprises of two branches: the main one, where the sensor under characterization is mounted on, and a second one which acts as a bypass for reducing the incoming flow from the centrifugal fan. The value of the desired flow is set by adjusting accordingly the needle valve (V1), which is situated before the prototype sensor and the by-pass section valve (V2) of the setup, and it is monitored continuously by the air-velocity measuring unit before the inlet of the sensor.

Concerning the main branch of the flow network, the Reynolds number Re [35] and the entrance length L_h,turbelent_ in the case of turbulent flow after which the flow is considered fully developed [36] are calculated by the following equations:

(1) Re=ρ·Vavg·Dμ,

(2) $$L_{h,turbulent}=D\cdot 1.359\cdot {Re}^{1/4}$$

where V_avg_ is the average fluid velocity (m/s); D is the hydraulic diameter (m) which is equal to the diameter of the circular channel employed (0.019 m) in the particular setup; ρ is the density of air (kg/m^3^); and μ is the dynamic viscosity of air (Pa·s) equal to 1.225 kg/m^3^ and 17.89·10^−6^ Pa·s accordingly, considering an ambient temperature of 15 °C and atmospheric pressure.

The air velocity range employed in the characterization of the device is from 0 to 20 m/s in accordance with the performed CFD studies [19] for the specific flow-shaping topology. The calculated airflow, Reynolds number, and entrance length L_h,turbelent_ for the specific air velocity range are presented in Table 3; from the obtained values, we can conclude that the developed flow inside the main branch is always turbulent since Re > 4000 [36], while the maximum calculated corresponding entrance length is equal to 0.33 m. According to [37], however, the entrance length can be significantly higher; more specifically, in a circular channel, for a turbulent flow to be fully developed, it can be equal to even forty diameters, i.e., L_h,turbelent_ = 40·D, which is equal to 0.57 m for the specific setup. For the above reasons, the length of the flow network before the sensor was set equal to one meter, which is significantly greater than the minimum required for the incoming flow to be developed.

### 3.3. Initial Electrical Characterization of the Developed Sensor

A preliminary electrical characterization of the prototype was performed employing the experimental setup discussed in the previous section for the airflow values presented in Table 3. Both cases of one Bluff Body and two Bluff Bodies with different combinations regarding their locations were investigated for the specific air velocity range, in accordance with [19]. In more detail, in the first case (Case A), the variation in the piezoelectric element’s resistance (ΔR) was monitored in response to the incoming airflow when one Bluff Body is situated at one of the three positions along the flow: p0, p4, and p6 (see Figure 2); consequently, in the second case (Case B), the resistance variation (ΔR) was monitored for the same flow range but for two Bluff Bodies present, situated at various combinations of positions. The configurations considered for both cases are presented in Table 4.

During the characterization experiments, a constant current was applied to the piezoresistive element of the sensor, while the developed voltage was monitored continuously in order to deduce the resulting resistance variation (ΔR). All the measurements were collected directly from the source meter, while no signal processing was applied. We should note at this point that the Temperature Coefficient of Resistance (TCR) for a CNT-enriched thermoplastic composite has been reported in the literature to have a value as high as −1.28 × 10^−2^/°C [25]; thus, even a small temperature increase due to self-heating of the element can potentially result in a significant decrease in its resistance. Bearing this in mind, a considerably low value of 12.5 μA was employed for the constant current applied during the electrical characterization. Note that the corresponding power dissipation for the specific meander type piezoresistor with a resistance of 49.25 kΩ and a volume of 21.63 mm^3^ (see Section 3.1 and Section 2.3, respectively) is 7.7 μW, which was considered to be small enough not to induce self-heating that would lead to a significant resistance decrease. The above was confirmed experimentally by monitoring the sensing element’s resistance and confirming that it remains consistent after a current of 12.5 μA is applied with no airflow present.

**Figure 8:**
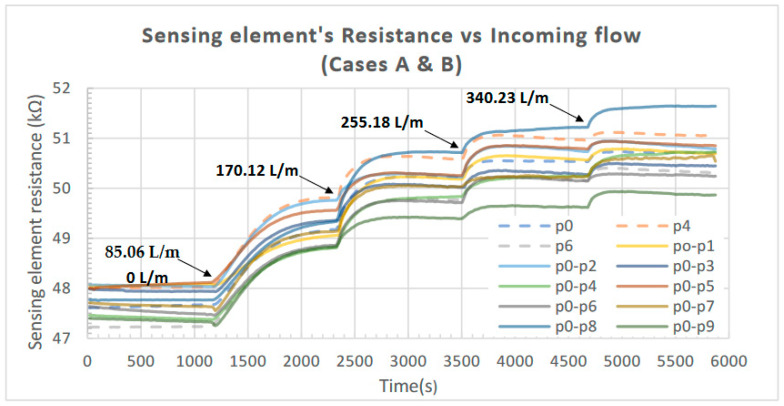
Measured resistance of the piezoelectric element in response to the incoming airflow for all the combinations regarding the location of the Bluff Bodies (Cases A and B).

**Figure 9:**
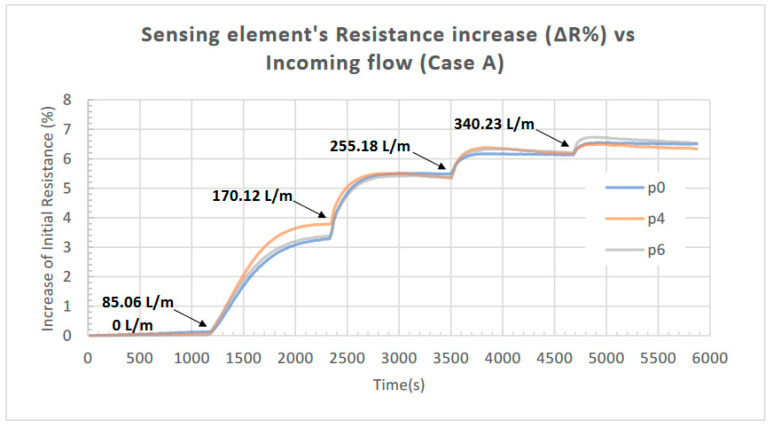
Increase in the piezoelectric element’s resistance (ΔR) as a percentage of its initial value in response to the incoming airflow for the three different Bluff Body positions: p0, p4, and p6 (Case A); a similar response is observed for the three positions.

**Figure 10:**
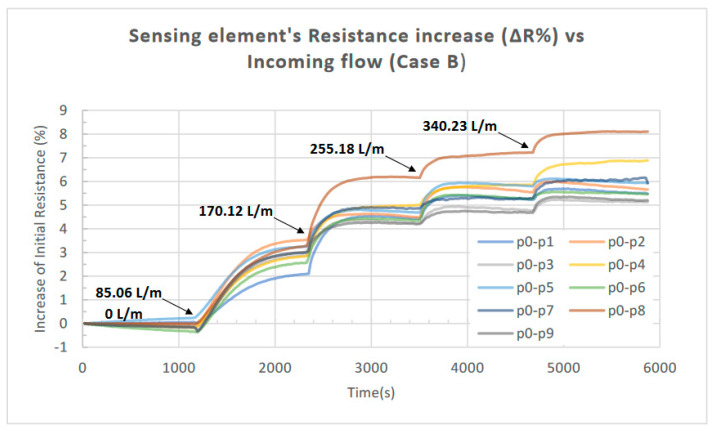
Increase in the piezoelectric element’s resistance (ΔR) as a percentage of its initial value in response to the incoming airflow for various combinations of two Bluff Bodies as presented in Table 4 (Case B); an enhanced response is noticed for the combination p0-p8.

The obtained results from the initial characterization of the device are presented in the following figures. The measured resistance of the piezoelectric element in response to the incoming airflow (for all the combinations regarding the location of the Bluff Bodies examined) is presented in Figure 8; the increase in the resistance (ΔR) as a percentage of its initial value is presented in Figure 9 for Case A (one Bluff Body), while the increase in response to the incoming airflow for Case B (two Bluff Bodies) is presented in Figure 10.

It is important to note that the same prototype device was employed for each of the configurations considered; therefore, the same 3D-printed piezoresistor was used for all the measuring cycles performed.

In all cases, the applied airflow increases sequentially from 0 to 340.23 L/min in four steps. Each step has a duration of 20 min (1200 s) and increases the airflow value by 85.06 L/min (the values of airflow applied at each step are noted in the corresponding figures).

**Figure 11:**
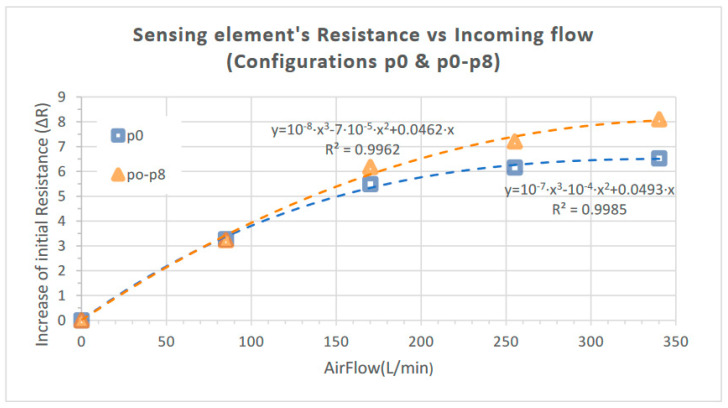
Increase in the piezoelectric element’s resistance (ΔR) as a percentage of its initial value versus the incoming airflow for the Bluff Body configurations p0 (Case A) and p0-p8 (Case B).

Based on the above results, the increase in the piezoelectric element’s resistance (ΔR) as a percentage of its initial value versus the incoming airflow is presented in Figure 11 for the Bluff Body combinations p0 and p0-p8 (Cases A and B, respectively). A third-degree polynomial fit (with R^2^ > 0.996) has been added in both cases in order to reflect the overall trend in the specific data.

## 4. Discussion

As a first and more comprehensive remark from the experimental results obtained, we should note that the proposed device’s proof-of-concept has been validated; the developed sensing device which is fabricated using exclusively 3D FDM technology and two commercially available building materials, can measure successfully an incoming airflow in the range from 0 to 350 L/min. In more detail, in both investigated cases (Cases A and B) regarding the Bluff Body (or Bodies) employed, an increase in the incoming airflow at the inlet of the flow shaping part corresponds to an increase in the maximum variation in the piezoresistor’s resistance (ΔR), indicating a monotonic relationship between the two factors. This is in accordance with a recently performed CFD study investigating the induced pressure variation for a similar flow range [38] considering the same flow channel, Bluff Body geometry and positions; therefore, it is safe to assume that Karman vortex street is formed for the specific airflow range due to the presence of the Bluff Body (or Bodies) in the flow shaping part of the prototype (Section 2.2), as predicted in [19] and [38] for the specific geometry.

The induced pressure evolution due to the formation of the Karman vortex street leads to an adequate deformation of the flexible free-standing membrane which is detected by the 3D-printed sensing element. The corresponding maximum increase in its initial resistance value is 6.68% and 8.1% when one Bluff Body and two Bluff Bodies are employed, respectively (Cases A and B), as presented in Figure 10, while saturation occurs at an airflow of approximately 350 L/m, as illustrated in Figure 11.

Regarding the printed sensing element, integrated on top of the free-standing membrane (both are printed in a single step), a significant piezoresistive response was recorded, as expected. For the mechanism governing the specific response, we should note the following: In reference [25], filament feedstocks were fabricated by melt blending of polypropylene random copolymer (PP-R) with multiwall carbon nanotubes (MWCNTs) using a twin-screw extruder; for all the predetermined amount of CNs investigated (4, 6, or 8 wt%) in the particular work, a piezoresistive response was observed that was attributed to the presence of CNs in the polymer matrix. In more detail, a tensile stress applied to CN-enriched polymer-based printed composites results in a corresponding tensile strain, which leads to an increase in the tunneling gaps between adjacent CNs in the polymer matrix; as a result, the composite’s resistance increases. Although the weight percentage (wt%) of CNs in the commercially available building material [23] that was employed in our study is unknown, the specific mechanism is in accordance with our results; thus, the measured resistance of the piezoelectric element increases due to the stress originating from the deformation of the membrane, for all the combinations regarding the location of the Bluff Bodies, in response to the incoming airflow (i.e., due to the induced pressure underneath when Karman vortex street forms).

As already presented in Table 4, twelve different configurations were investigated regarding the number and combinations of the positions of the Bluff Bodies. According to the relevant simulation CFD study performed in [19], the use of two Bluff Bodies positioned in a series along the flow stream significantly increases the maximum pressure variation induced, compared to that caused by one Bluff Body. Since we recorded the resulting variation in the piezoresistor’s resistance (ΔR) on top of the diaphragm and not the deduced pressure due to the formation of Karman vortex street, a direct comparison between the reported simulation results and those experimentally obtained in our study is not possible. However, the current work verifies the reported findings in the literature [19] since a considerable increase in ΔR was found in Case B compared to Case A. In more detail, ΔR was found to be 6.68% and 8.1% of the initial resistance for Cases A and B, respectively; therefore, the recorded resistance variation at the maximum incoming airflow is increased by 21.3% when two Bluff Bodies are situated in the flow stream at the optimum configuration with regard to that caused when one Bluff Body is employed.

According to the results presented in Figure 9, the location of one Bluff Body in the flow-shaping part (i.e., p0, p4, or p6) does not significantly influence the response of the proposed device. This outcome is expected since the resulting pressure deviation should not be affected by the exact position of the single Bluff Body along the flow channel, as long as there is an adequate channel length after the Bluff Body for the Karman vortex street to be fully developed. The slight variations observed in the three responses in Figure 9 can be attributed to the marginally different tensile strain developed on the piezoresistor for each case since its position remains fixed with respect to the channel inflow for all the Bluff Body positions investigated. In contrast, as we can observe in Figure 10, in the case of employing two Bluff Bodies (case B), the combination with regard to their locations along the flow stream has a substantial impact on the response of the device. In more detail, the combination p0-p8 leads to an 8.1% increase in the piezoresistor’s initial resistance compared to 5.3% for the combination p0-p3. Although there is no available CFD study to support the above finding, it can be primary attributed to the resulting maximum pressure deviation (ΔP) induced by the formation of the Karman vortex street for the particular topologies of Bluff Bodies.

## 5. Conclusions

In the present study, a new state-of-the-art manufacturing procedure for the development of a novel monolithic sensing device employing only 3D-printing FDM technology is presented. The device’s principle of operation is based on vortex shedding, and it can be manufactured using a commercially available desktop 3D printer and building materials fast, on-site, and on demand, contributing to a reduction in the extent of the required stock-keeping onboard regarding safety critical equipment/spare parts.

The proposed device’s structure comprises two main parts, a flow shaping part, responsible for the development of vortex shedding, and a sensing part, a printed piezoresistor integrated on top of an appropriate diaphragm for the detection of the deduced pressure deviation due to the formed Karman vortex street. The geometry of the flow-shaping section is based on previous research work regarding the development of a pneumatic energy generator using vortex shedding and CFD parameterization of its geometry considering a fixed incoming air velocity.

The effect of low-temperature thermal sintering on the electrical resistance of the conductive piezoresistive 3D-printed element was investigated, revealing its important role in the resulting value of the obtained resistance, in correspondence with the cases of inkjet printing or screen-printing. Note that, due to the limitation imposed by the building materials, regarding the applied temperature, a significantly larger time interval is required.

An initial electrical characterization of the developed sensor was performed, employing an appropriate experimental setup validating the design’s proof-of-concept. Briefly, the characterization was performed for various combinations concerning the location of a single Bluff Body or two Bluff Bodies, along the flow stream, underneath the sensing part of the device. According to the obtained results, the developed prototype can successfully detect an incoming airflow in the range from 0 to 350 L/min, confirming that the induced pressure evolution due to the formation of the Karman vortex street leads to an adequate deformation of the flexible free-standing membrane which is detected by the sensing element.

Twelve different configurations were investigated during the performed characterization, regarding the number and the combinations for the positions of the Bluff Bodies. In summary, the use of two Bluff Bodies positioned in series along the flow stream can significantly increase the maximum pressure variation induced, compared to that caused by a single one. Furthermore, the location of a single Bluff Body in the flow-shaping part does not markedly influence the response of the proposed device, while in the case of employing two Bluff Bodies, the combination with regard to their locations along the flow stream has a substantial impact.

Concerning the 3D-printed sensing element, a notable piezoresistive response (attributed to the presence of CNs in the polymer matrix of the filament) was recorded as expected; the variation in its initial resistance for the maximum airflow is within the range from 5.3% to 8.1% depending on the number and the topology of the Bluff Bodies. It is worth noting that all the elements comprising the sensing part of the prototype are printed through additive manufacturing in a single step; that is, the rigid frame supporting the diaphragm, the piezoresistor, the vias, the electric pads, and the diaphragm itself consist a single printed part.

Furthermore, direct communication to the necessary readout circuitry can be easily achieved through standard soldering employing the integrated contact pads, with immediate benefits in the device’s complexity, mechanical reliability, process time, and cost. In addition, the developed prototype can be considered as a 4D-printed stimuli-responsive device since both the flexible membrane and the sensing element, integrated on top, considerably alter their form in response to the appropriate stimuli, i.e., the presence of flow.

This study could contribute to the investigation of fabricating printed sensing devices employing solely FDM additive manufacturing, novel design, and commercially available building materials, with immediate benefits such as low cost, fast on-site manufacturing of the complete measuring device, robustness, and simplicity, suggesting numerous potential industrial applications which are obviously not limited to the maritime sector. Future work includes a comprehensive study of the mechanical properties of the building materials employed, an investigation of the piezoresistive response of the 3D-printed piezoresistor in relation to its design, the CN-enriched building materials, and the infill pattern employed.

## Figures and Tables

**Table 1:** Main characteristics for the conductive filament FiberForce Nylforce CNT Conductive.

Main Characteristics	Value
Density	1.35 g/cm^3^
Melting Point	145–160 °C
Tensile Strength	30 MPa
Elastic Modulus	1550 MPa
Surface Electric Resistivity	10 Ω/sq

**Table 2:** Printing parameters for both materials employed for the fabrication of the prototype.

Printing Parameters	FiberForce Nylforce CNT Conductive	BASF Acrylonitrile Styrene Acrylate (ASA)
Nozzle Temperature	260	215
Nozzle Diameter	0.6 mm	0.4 mm
Nozzle Material	Brass	Brass
Printing Speed	45 mm/s	45 mm/s

**Table 3:** Air-velocity values employed in the characterization of the device and the corresponding airflow, Reynolds number, and entrance length for the flow network employed. Note that the developed flow is always turbulent while the maximum entrance length derived is equal to 0.33 m.

Air Velocity (m/s)	Airflow (L/min)	Reynolds Number	Entrance Length (m)
0	0	0	0
5	85.06	6505.03	0.23
10	170.12	13010.06	0.28
15	255.18	19515.09	0.31
20	340.23	26020.12	0.33

**Table 4:** Different configurations concerning the number and the location of Bluff Body (or Bodies) that were investigated under the same airflow conditions.

Bluff Bodies Configuration	Location(s) of Bluff Body (or Bodies)
Case A (One Bluff Body employed)	p0; p4; p6
Case B (Two Bluff Bodies employed)	p0-p1; p0-p2; p0-p3; p0-p4; p0-p5; p0-p6; p0-p7; p0-p8; p0-p9

## Data Availability

The data presented in this study are available on request from the corresponding author.
